# Infectious Spleen and Kidney Necrosis Virus ORF093R and ORF102R Regulate Glutamate Metabolic Reprogramming to Support Virus Proliferation by Interacting with c-Myc

**DOI:** 10.3390/ijms26020718

**Published:** 2025-01-16

**Authors:** Yinjie Niu, Caimei Ye, Qiang Lin, Hongru Liang, Xia Luo, Baofu Ma, Ningqiu Li, Xiaozhe Fu

**Affiliations:** Ministry of Agriculture and Rural Affairs, Key Laboratory of Aquatic Animal Immune Technology, Key Laboratory of fishery Drug Development, Pearl River Fisheries Research Institute, Chinese Academy of Fishery Sciences, Guangzhou 510380, China; niuyinjie0530@163.com (Y.N.); nancyye95@163.com (C.Y.); hrliang13@126.com (H.L.); lxwenhao@163.com (X.L.); mabf@prfri.ac.cn (B.M.)

**Keywords:** ISKNV, c-Myc, glutamine metabolism

## Abstract

Glutamine metabolism is essential for infectious spleen and kidney necrosis virus (ISKNV) replication. Glutaminase 1 (GLS1), the key enzyme of the glutamine metabolism, and c-Myc positively regulate ISKNV infection, while c-Myc is closely correlated with GLS1. However, the regulatory mechanism among ISKNV, c-Myc and glutamine metabolism remains unclear. Here, we indicated that c-Myc increased glutamine uptake by increasing the GLS1, glutamate dehydrogenase (GDH) and isocitrate dehydrogenase (IDH2) expression of glutamine metabolism. ISKNV ORF102R, ORF093R and ORF118L co-located with c-Myc in CPB cells. Co-IP results showed that ISKNV ORF102R and ORF093R interacted with c-Myc, while ORF118L did not interact with c-Myc. The expression levels of c-Myc, GLS1 and IDH2 were increased in ISKNV ORF093R expression cells, and the mRNA and protein levels of GLS1 were upregulated in ISKNV 102R-expressing cells. These results indicated that ISKNV reconstructed glutamine metabolism to satisfy the energy and macromolecule requirements for virus proliferation by ORF093R and ORF102R interacting with c-Myc, which provides the foundation for innovative antiviral strategies.

## 1. Introduction

Infectious Spleen and Kidney Necrosis Virus (ISKNV) is the main pathogen causing explosive diseases in mandarin fish [1]. The increasing frequency of ISKNV outbreaks is severely hindering the green and healthy development of the mandarin fish aquaculture industry [2]. Glutamine is a conditionally amino acid which supports the rapid growth and proliferation of cancer cells [3]. Our previous studies found that glutamine and glutamine metabolism are essential for ISKNV replication and proliferation [4]. GLS1 is the key enzyme of glutamine metabolism; its inhibitor, BPTES, inhibits ISKNV replication and proliferation by reducing GLS activity [4]. ISKNV infection increases GLS1 and c-Myc expression, while GLS1 and c-Myc positively regulate ISKNV replication and proliferation [5,6,7]. c-Myc is reported to regulate glutamine metabolism by increasing GLS expression [3,8,9]. It can upregulate GLS1 via miR-23a/b-dependent post-transcriptional mechanisms or by directly binding to the GLS1 transcription start site [9,10]. These results suggest that ISKNV may regulate glutamine metabolism via c-Myc.

Viruses can manipulate cellular glutamine metabolism by c-Myc. Kaposi’s Sarcoma-associated Herpesvirus (KSHV) activates the Myc/MondoA-network to increase glutamine uptake for glutaminolysis [11]. Influenza infection induces an increase in c-Myc, which may contribute to glutamine metabolism requirements for infected NHBE cell survival [12]. c-Myc promotes glutamine conversion to ketoglutarate, resulting in cellular addiction to glutamine in human cytomegalovirus (HCMV)-infected cells [13,14,15]. E4ORF1 interaction with c-Myc reprograms glutamine metabolism by regulating the expression of glutamine transporters SLC7A5/ASCT2 and SLC1A5/LAT1 [14,16]. However, the regulatory mechanisms among ISKNV, c-Myc and glutamine metabolism remain unclear.

**Figure 1:**
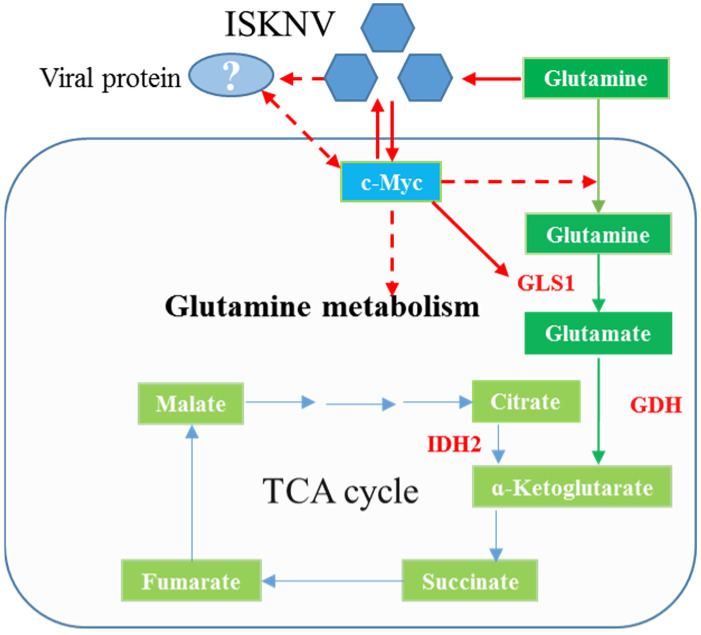
A model diagram of the problem to be solved in this research.

In this study, we explored the effect of c-Myc on key enzymes of glutamine metabolism in CPB cells and searched for the viral proteins interacting with c-Myc to regulate glutamine metabolism (Figure 1). We found that c-Myc positively regulates GLS1, GDH and IDH2 expression levels. ISKNV ORF093R and ORF102R both interact with c-Myc. c-Myc, GLS1 and IDH2 expression levels are upregulated in ORF093R expression cells, while c-Myc, GLS1 mRNA and protein levels are increased in ORF102R expressed cells. These results indicate that ISKNV regulates glutamine metabolism for virus replication by ORF093R and ORF102R interacting with c-Myc.

## 2. Results

### 2.1. c-Myc Positively Regulates Glutamine Metabolism

**Figure 2:**
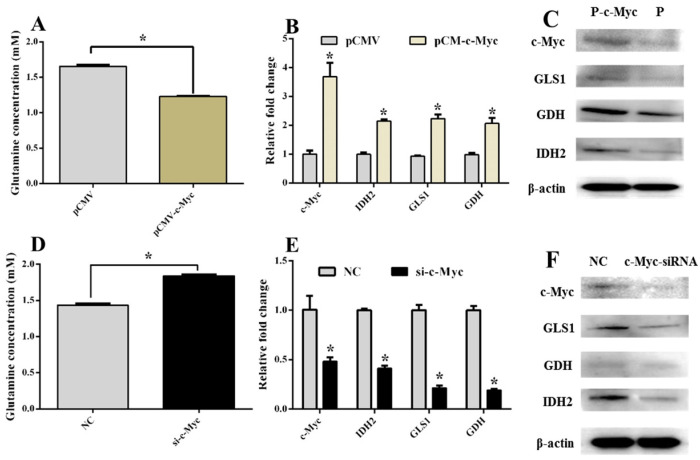
c-Myc positively regulated glutamine metabolism. (**A**) The concentration of glutamine was significantly downregulated in the supernatant of c-Myc overexpression cells. (**B**) Overexpression of c-Myc increased IDH2, GLS1 and GDH mRNA expression. (**C**) The c-Myc, IDH2, GLS1 and GDH protein levels were markedly increased in c-Myc overexpression cells. (**D**) Glutamine was significantly decreased in cells transfected with si-c-Myc. (**E**) c-Myc, IDH2, GLS1 and IDH2 mRNA expression levels were significantly upregulated in cells transfected with si-c-Myc. (**F**) The IDH2, GDH and GLS1 protein expression levels were markedly decreased in knocked-down c-Myc. *: *p* < 0.5; *p* < 0.05 represents a statistical difference.

ISKNV infection promotes c-Myc expression [5]. c-Myc increases glutamine uptake by targeting GLS1 and IDH2 expression in glutamine metabolism [17], which implies that c-Myc regulates glutamine metabolism in ISKNV-infected cells. To elucidate the influence of c-Myc on glutamine uptake and glutamine metabolism in ISKNV-infected cells, we used c-Myc overexpression cells and synthesized c-Myc-siRNA. Glutamine uptake was significantly increased in c-Myc overexpression cells; however, knocking down c-Myc significantly reduced glutamine uptake (Figure 2A,D). Overexpressing c-Myc significantly increased GLS1, GDH and IDH2 mRNA (Figure 2B), and GLS1, GDH and IDH2 protein expression levels were significant in overexpression cells (Figure 2C). GLS1, GDH and IDH2 mRNA expression levels were dramatically downregulated in knocked-down endogenous c-Myc cells (Figure 2E), while GLS1, GDH and IDH2 protein expression levels were significantly decreased in knocked-down c-Myc cells. These results indicated that c-Myc regulated glutamine metabolism to support virus replication by increasing the key enzymes of GLS1, GDH and IDH2 expression.

### 2.2. Screening the ISKNV Proteins’ Interaction with C-Myc

Protein samples of ISKNV-infected cells were collected and subjected to IP by using a c-Myc antibody. Mass spectrometry results indicated that six viral proteins were identified in c-Myc IP samples, namely ORF085R, ORF087R, ORF111R, ORF118L, ORF102R and ORF093R. The peptide intensity values of ORF118L, ORF102R and ORF093R were relatively high (Table 1).

**Figure 3:**
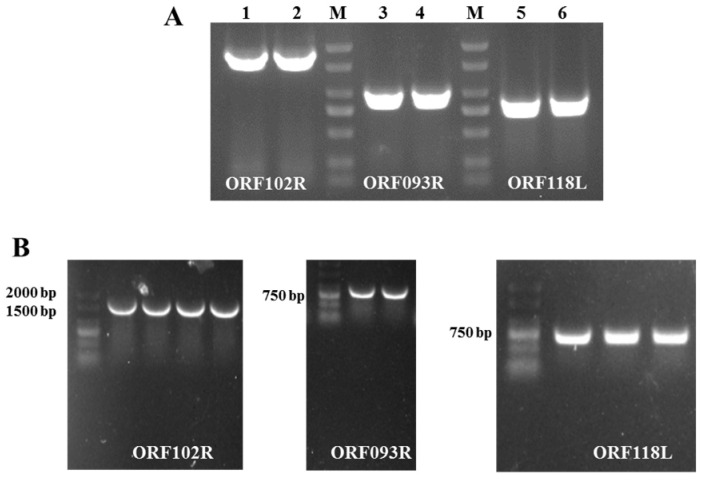
pcDNA-ORF102R-Flag, pcDNA-ORF093R-Flag and pcDNA-ORF118L-Flag were constructed. (**A**) The corresponding fragments of ORF102R, ORF093R and ORF118L were amplified by PCR. (**B**) The positive clones of pcDNA-ORF102R-Flag, pcDNA-ORF093R-Flag and pcDNA-ORF118L-Flag were identified by PCR.

PCR reactions were used to obtain ISKNV ORF102R, ORF093R and ORF118L gene fragments with a Flag tag sequence (Figure 3A). Those PCR products were recovered and ligated to pcDNA3.1 (+). The positive clones were identified by PCR reactions and sequencing (IGE Technology, Guangzhou, China) (Figure 3B). These results showed that pcDNA-ORF102R, pcDNA-ORF093R and pcDNA-ORF118L vectors were successfully constructed.

### 2.3. ORF102R, ORF093R and ORF118L Co-Located with C-Myc

**Figure 4:**
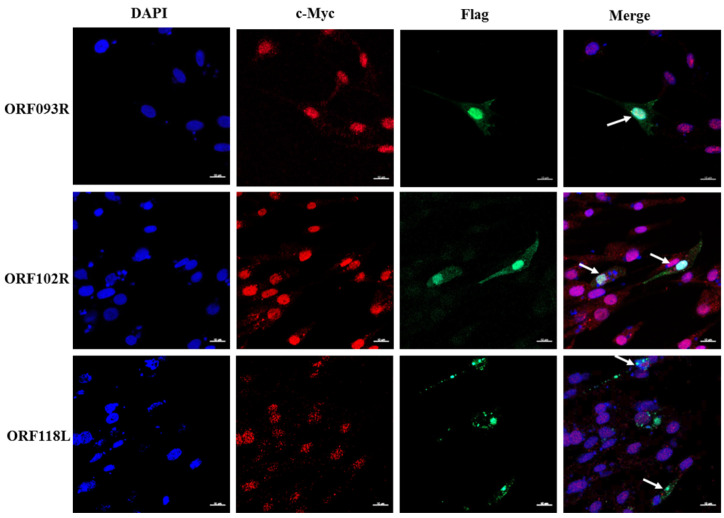
ORF102R, ORF093R and ORF118L co-located with c-Myc. The ORF093R and ORF102 expression levels were completely overlapped with c-Myc, but the ORF118L expression was partially overlapped with c-Myc. C-Myc showed red fluorescence, Flag showed green fluorescence, “—” represented the scale bar of 10 μm, The white arrow represented the overlap.

ORF102R, ORF093R and ORF118L vectors were transfected into CPB cells by using EZ 3000 transfection. Cofocal microscopy results showed that ORF118L exhibited a certain degree of intracellular co-localization with c-Myc, while ORF102R and ORF093R expression showed complete overlap with c-Myc (Figure 4). This suggested that there may be interactions between ISKNV ORF102R, ORF093R and ORF118L proteins and c-Myc.

### 2.4. ISKNV ORF102R and ORF093R Interacted with c-Myc

**Figure 5:**
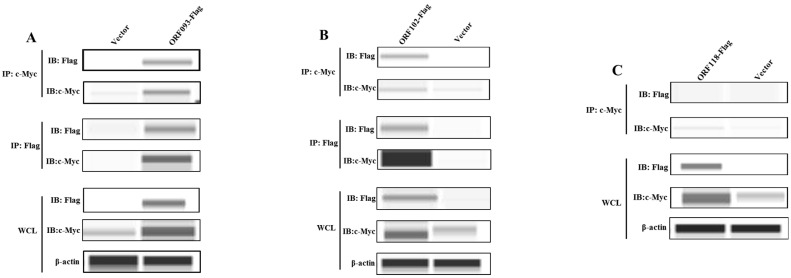
ORF102R and ORF093R interacted with c-Myc, but ORF118L did not interact with c-Myc. (**A**–**C**) After transfection for 48 h, the cells were lysed and immunoprecipitated with the anti-Flag or anti-c-Myc antibody.

To further confirm that ISKNV ORF102R, ORF093R and ORF118L interacted with c-Myc, a Flag monoclonal antibody and c-Myc polyclonal antibody were used for a Co-IP assay. The c-Myc antibody could detect ORF093 and ORF102 proteins in ORF102R and ORF093R IP samples, and c-Myc in the two IP samples was detected by using the Flag antibody (Figure 5A,B). However, c-Myc could not detect 118L proteins, and the Flag antibody did not detect c-Myc protein in the ORF118L IP sample (Figure 5C). These results indicated that ORF102R and ORF093R interacted with c-Myc, but ORF118L did not interact with c-Myc.

### 2.5. The Effect of ISKNV ORF093R, ORF102R and ORF118L on Glutamine Metabolism

To further investigate the ISKNV-regulated glutamine metabolism through ORF093R, ORF102R and ORF118L, we detected the c-Myc, GLS1, GDH and IDH2 mRNA and protein expression in ORF093R, ORF102R and ORF118L overexpression cells.

**Figure 6:**
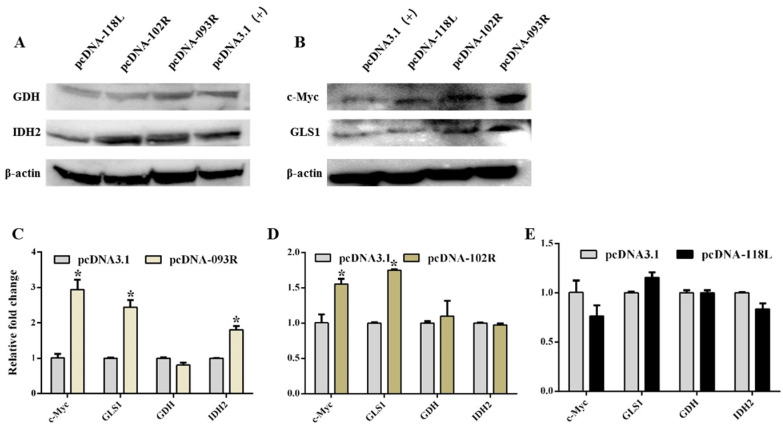
The effect of ISKNV ORF093R, ORF102R and ORF118L on glutamine metabolism. (**A**) The expressions of GDH and IDH2 protein levels were significantly upregulated in ORF093R-transfected cells, and the IDH2 protein levels were markedly upregulated in ORF102R-transfected cells. (**B**) The c-Myc protein levels were markedly upregulated in cells transfected with ORF093R, ORF102R and ORF118L, and the GLS1 proteins were significantly increased in cells transfected with ORF093R and ORF102R. (**C**) The mRNA expression levels of c-Myc, GLS1 and IDH2 were upregulated in cells transfected with pcDNA-093R. (**D**) The c-Myc and GLS1 mRNA were increased in cells transfected with pcDNA-102R. (**E**) The mRNA expression levels of c-Myc, GLS1, GDH and IDH2 showed no change in cells transfected with ORF118L. *: *p* < 0.5; *p* < 0.05 represents a statistical difference.

ORF093R significantly increased c-Myc, GLS1, and IDH2 mRNA expression (Figure 6C). c-Myc, GLS1, and IDH2 protein expression were significantly increased in ORF093R-expressing cells, but GDH mRNA and protein expression showed no change in ORF093R-expressing cells (Figure 6A,B). This suggested that ISKNV ORF093R regulated the host cell’s glutamine metabolism through upregulating c-Myc, GLS1 and IDH2 mRNA and protein expression.

qRT-PCR indicated that GLS1 and c-Myc mRNA expression levels were significantly upregulated (Figure 6D); however, GDH and IDH2 mRNA expression showed no significant change in CPB cells transfected with ORF102R cells. The WB results indicated that GLS1 and c-Myc protein expression levels were significantly increased; however, GDH and IDH2 protein levels showed no significant change in ORF102R-expressing cells (Figure 6A,B). This suggested that ORF102R interacts with c-Myc, which promoted glutamine metabolism to increase GLS1 expression. We speculated that the interaction between ORF102R and c-Myc promoted c-Myc binding to GLS1 promoter, which upregulated GLS1 expression and glutamine metabolism in the ISKNV-infected cells.

qRT-PCR indicated that GLS1, GDH and IDH2 mRNA expression showed no change, while c-Myc mRNA expression was upregulated in ORF118L-expressing cells (Figure 6E). The WB results showed that protein levels of c-Myc were significant in cells transfected with ORF118L; however, the mRNA levels of GLS1, GHD and IDH2 showed no change in cells transfected with ORF118L, which was consistent with the changes in mRNA expression (Figure 6A,B). This result indicated that ISKNV ORF118L did not affect the glutamine metabolism.

## 3. Discussion

Glutamine is a major metabolic fuel for energy and nitrogen for biosynthesis in proliferating cells [18,19]. Viruses alter glutamine metabolic pathways for optimal virion production [20,21]. Our previous study indicated that glutamine deprivation decreased ISKNV infection in CPB cells, and GLS1 and its activity played an important role in ISKNV infection [5,7]. In this study, we found that c-Myc increased the uptake of glutamine and the GLS1, GDH and IDH2 expression of glutaminolysis. ISKNV ORF093R and ORF102R regulated GLS1 and IDH2 enzymes of glutamine metabolism by interacting with c-Myc. These results suggested that ISKNV increased GLS1 and IDH2 expression to regulate glutamine metabolism by ORF093R and ORF102R interacting with c-Myc.

The glutamine consumption was increased in host cells infected with HCMV, HSV or VACV [21,22,23]. Our previous study showed that the ISKNV-infected cells developed glutamine addiction for virus replication [4]. c-Myc induces glutamine addiction for cells’ survival and growth in different tumors [24]. MYC activation increases the reductive glutamine carboxylation in cells infected with adenovirus [16]. mRNA and protein levels of c-Myc were significantly upregulated in ISKNV-infected cells [5]. Here, we demonstrated that overexpressing c-Myc promoted the uptake of glutamine into CPB cells. These results suggested that ISKNV infection increased the glutamine uptake by elevating c-Myc expression.

Glutamine is catalyzed into glutamate by the glutaminase 1 (GLS1) enzyme; then, glutamine dehydrogenase (GDH) catalyzes the conversion of glutamate into α-ketoglutarate (α-KG) [25,26,27]. When the α-KG is carboxylated to isocitric acid by isocitrate dehydrogenase (IDH), it enters reductive glutamine metabolism (RGM) [28]. ISKNV infection increases the mRNA and protein of GLS1 and c-Myc [4,7]. c-Myc is demonstrated to stimulate glutamine metabolism by upregulating GLS1 to support the growth of cancer cells [19]. c-Myc binding to the IDH2 promoter promoted IDH2 transcription, regulating the RGM [29]. In this study, GLS1, GDH and IDH2 mRNA and protein expression were upregulated in overexpressing c-Myc cells. The overexpression of c-Myc and GLS1 promoted ISKNV infection. These results indicate that ISKNV regulates the RGM of glutamine metabolism via c-Myc.

Viruses increase glutamine metabolism to support self-replication through using viral proteins. KSHV k1 promoted c-Myc expression by binding to c-Myc. The ADV E4ORF1 protein reprogrammed glutamine metabolism by interacting with c-Myc [11]. In this study, ISKNV ORF093R significantly increased c-Myc and GLS1 and IDH2 expression by interacting with c-Myc, but there was no change in GDH expression. This result indicated that there may be other ISKNV proteins regulating glutamine metabolism. Latent membrane protein 1 (LMP1) of Epstein–Barr virus (EBV) enhanced IDH2 transcription by promoting c-Myc binding to the IDH2 promoter. ISKNV ORF102R interacting with c-Myc promoted c-Myc and GLS1 expression, which is consistent with the norovirus NS1/2 protein enhancing GLS enzyme activity to increase glutamine metabolism [20]. Meanwhile, ORF102 is predicted as the repeat sequence anchoring protein, which mainly mediates protein–protein interaction [30]. These results suggest that ORF102 may promote c-Myc binding to GLS1 upregulating GLS1 transcription and expression. The above data indicate that ISKNV could regulate glutamine metabolism by ORF093R and ORF102R interacting with c-Myc.

## 4. Materials and Methods

### 4.1. Cells and Viruses

The CPB cells were established from *Siniperca chuatsi* brains and cultured in 8% FBS L-15 medium in our lab [31]. The ISKNV strains was isolated in our lab [32]. The cells of overexpressing c-Myc were constructed in our lab, and the si-c-Myc used was synthesized in our lab [5].

### 4.2. RNA Extraction and Relative Fluorescence Quantification PCR

The total mRNA of CPB cells was extracted with Trizol reagent (Invitrogen, Waltham, MA, USA). The reverse transcription of mRNA into cDNA was carried out with a PrimeScript RT reagent kit with gDNA Eraser (Takara, Tokyo, Japan). The quantitative qRT-PCR was performed using SYBR premixed Ex Taq (Takara, Tokyo, Japan). The housekeeping gene was 18S, and the used primers are listed in Table 2. The relative change was calculated by using the 2^−ΔΔCT^ method.

### 4.3. Cell Protein Sample Preparation and Western Blotting

The CPB cells were collected in 1.5 mL centrifuge tubes. To the cell pellet, lysis buffer RIPA (Radio Immunoprecipitation Assay, RIPA) containing 1% protease inhibitor (PMSF) as added. Then, the SDS loading buffer was added into the protein supernatant and boiled for 10 min, and the prepared cell protein samples were stored at −80 °C.

The protein samples were electrophoretically separated in 12% SDS-PAGE gel and then transferred to PVDF membrane by using a Wet Mold Rotator (Mini Trans-Blot Electrophoretic Transfer Cell, Bio-Rad, Hercules, CA, USA). The transferred membrane was blocked in 3% BSA and incubated with primary antibodies for 2 h. After being washed three times, the membrane was incubated with HRP-conjugated second antibody (1:4000) at room temperature for 1 h. The bands were visualized using ECL (ComWin Bio, Beijing, China) according to the manufacturer’s instructions.

### 4.4. Immunoprecipitation and Mass Spectrometry

The steps of immunoprecipitation were performed as follows: the CPB monolayer cells in a T75 flask were incubated with ISKNV and SCRV for 12 h and 48 h, respectively. The protein sample was collected as described in Section 4.3, and the protein supernatant was incubated with 1 μg of c-Myc antibody on a 3D shaker overnight; 30 μL of protein A/G beads was washed three times with 1 mL of RIPA (without PMSF), and the protein A/G beads and cell–antibody mixture were incubated at 4 °C overnight.

The samples were sent to the New Life Company (Shanghai, China) for mass spectrometry analysis. Briefly, the following steps were used: the protein bands were digested with sequencing-grade trypsin and desalted on a C18 cartridge (Empore™ SPE Cartridges C18, St. Paul, MN, USA), and the peptides were enriched by using the High-SelectTM Fe-NTA Phospho Enrichment Kit (Thermo Scientific, Waltham, MA, USA) and analyzed on the timesTOF Pro mass spectrometer (Bruker, Karlsruhe, Germany); then, the raw mass spectrometry data of each sample were identified and analyzed by using MaxQuant software (http://www.maxquant.org/).

### 4.5. Eukaryotic Plasmid Construction and Fluorescence Colocalization

Based on the proteomic analysis, ISKNV ORF118L, ORF102R and ORF093R may interact with Sc-c-My. Primers were designed according to ISKNV (NC_003494.1, Table 2). The PCR products were double digested and ligated to the vector pcDNA3.1 (+). ISKNV ORF118L, ORF102R and ORF093R expression vectors with Flag tags were constructed.

CPB monolayer cells were transfected with pcDNA-ORF102R, pcDNA-ORF093R and pcDNA-ORF118L plasmids onto cell culture slides using Transfect EZ 3000 (Zeta life, San Francisco, CA, USA). Briefly, steps were performed as follows: the cells of 2.0 × 10^5^/mL were cultured in cell culture slides, and after 24 h of cultivation, the 10 μL 200 ng/μL vectors were mixed with 10 μL of transfection reagent, which was incubated at room temperature for 15 min, and then, the transfection complex was added to the cell slides. Then, 24 h post-transfection, the cells were fixed with 4% paraformaldehyde for 15 min. The cell slides were incubated with the Flag monoclonal antibody at a dilution of 1:2000 (Proteintech, Wuhan, China) and c-Myc polyclonal antibody at a dilution of 1:200 (our lab). The secondary antibodies conjugated with red and green fluorescent dyes at a dilution of 1:500 were added (Cwbio, Taizhou, China). Finally, the cells were observed by using a laser confocal microscope(Olympus, Tokyo, Japan).

### 4.6. Co-IP

The cell samples transfected with pcDNA-ORF102R, pcDNA-ORF093R and pcDNA-ORF118L vectors were collected. A c-Myc polyclonal antibody (1:100) and Flag monoclonal antibody (1:1000) were used to prepare IP samples. The loading buffer of the Wes isolation kit (ProteinSimple, San Jose, CA, USA) was added into IP samples and heated at 95 °C for 5 min; then, the prepared samples, Wes antibody diluent (ProteinSimple), primary antibody (Flag 1:2000, c-Myc 1:200), secondary antibody (ProteinSimple), luminol-peroxide, and washing buffer were added into the microplate (ProteinSimple), and the microplate underwent automated protein immunoblotting by using a Wes machine (ProteinSimple, San Jose, CA, USA).

### 4.7. Statistical Analysis

The relative change was calculated as the means ± standard deviation (SD) from at least 3 experiments. Statistical data were analyzed by one-way analysis of variance (ANOVA) (expressed as mean + SD). *p* < 0.05 represented the significance level. All data were compared using SPSS 13.0 (SPSS, Chicago, IL, USA).

## 5. Conclusions

**Figure 7:**
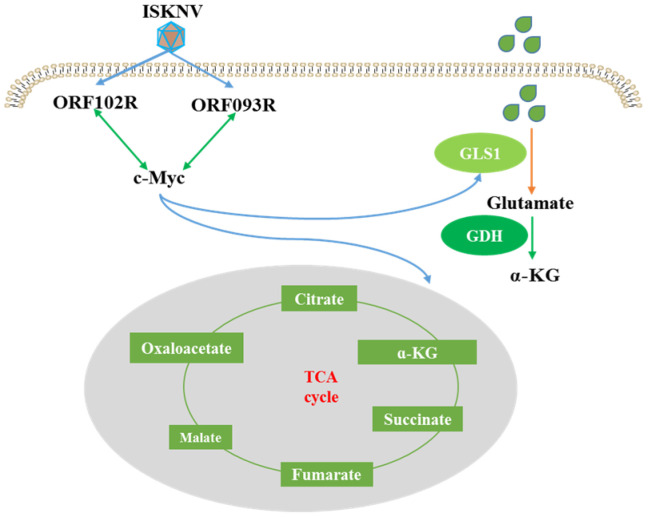
ISKNV can increase c-Myc, GLS1 and IDH2 to regulate glutamine metabolism via ORF093R and ORF102R interacting with c-Myc.

In this study, we explored the relationship between c-Myc and glutamine metabolism in ISKNV infection and explored viral proteins interacting with c-Myc to regulate glutamine metabolism. c-Myc increased those key enzymes of glutamine metabolism to promote ISKNV proliferation. ISKNV ORF093R upregulated c-Myc, GLS1 and IDH2 expression by interaction with c-Myc, while ISKNV ORF102R interacting with c-Myc only promoted the GLS1 expression. These results indicate that ISKNV can increase c-Myc, GLS1 and IDH2 to regulate glutamine metabolism for virus replication via ORF093R and ORF102R (Figure 7). Those findings raise the scientific problems of follow-up research and provide novel chokepoints for new therapeutic treatments for ISKNV infection.

## Figures and Tables

**Table 1:** ISKNV viral proteins were identified in c-MycIP samples.

Protein IDs	Intensity	MW (kDa)	Predicted	Description
E2CU38	68,329	53.68	102R	Ankyrin repeat-containing
Q4KS43	624,010	25.3	118L	Novel viral envelope
Q8QUM5	7561.5	22.65	085R	Unknown
M1SWU3	14,918	28.95	087R	Ribonuclease III family
Q5YF04	221,650	32.7	093R	Transmembrane protein
E2CU56	26,090	32.7	111R	C3HC4 type

**Table 2:** Primers used for constructing viral protein eukaryotic expression vectors.

Primer Name	Sequence(5′–3′)
OFR 102R-F	(KpnI) CG*GGGTAC*CGCCACCATGGCAGCAAACCAGACCATTG
OFR 102R-R	(BamHI) CGC*GGATCC*TTA CTTATCGTCGTCATCCTTGTAATCCGTTCCGCGCACACAAG (56 °C)
OFR 093R-F	(KpnI) CG*GGGTAC*CGCCACCATGACATCGAGGCATACATCAGACGC
OFR 093R-R	(BamHI) CGC*GGATCC*TTA CTTATCGTCGTCATCCTTGTAATCGAGCCGTATGGACTTGTGC (58 °C)
OFR 118L-F	(KpnI) CG*GGGTAC*CGCCACCATGGCATTCGCTACTACAATGAGAGT
OFR 118L-R	(EcoRI) CG*GAATTC*TTA CTTATCGTCGTCATCCTTGTAATCCACTTGACCAAAGAGGTCCT (56 °C)
18S-F	CATTCGTATTGTGCCGCTAGA
18S-R	CAAATGCTTTCGCTTTGGTC
c-Myc	ACCGCCACCTTCATCCTCTTCC (MN759310.1)
c-Myc	CGCCGCTCCTCCTCATCCTC
GLS1-F	TCCTGCGGCATGTACGACTTCT (MN267067)
GLS1-R	CCAGCTTGTCCAGTGGAGGTGA
GDH-F	AGGTCCGTCACTATGCCGATGC
GDH-R	AGATCCTCCACCAGCTTGTCCTC
IDH2-F	GTCATCAGTGTGGTCACGGTACG
IDH2-R	TGGAGATGGACGGAGACGAGATG

Note: the italic is the cleavage site, and the underline is the Flag label sequence.

## Data Availability

The data presented in this study are available on request from the corresponding author.
